# COSMO-RS guided screening of deep eutectic solvents and ultrasound-assisted extraction for resveratrol from *Polygonum cuspidatum*

**DOI:** 10.1016/j.ultsonch.2026.107795

**Published:** 2026-02-24

**Authors:** Yitong Lu, Zimeng Song, Pengjun Chen, Zunlai Sheng, Zhiyong Wu

**Affiliations:** a College of Veterinary Medicine, Northeast Agricultural University, Harbin 150030, PR China; b Heilongjiang Key Laboratory for Animal Disease Control and Pharmaceutical Development, Harbin 150030, PR China

**Keywords:** *Polygonum cuspidatum*(*P.cuspidatum*), Resveratrol, COSMO-RS, Ultrasound-assisted extraction (UAE), Deep eutectic solvent (DES)

## Abstract

Resveratrol is a phenolic compound renowned for its anti-inflammatory, antioxidant, and anti-apoptotic properties. To develop a greener alternative to conventional organic solvents, this research established an effective ultrasound-assisted extraction (UAE) approach utilizing deep eutectic solvents (DES) to isolate resveratrol from *Polygonum cuspidatum.* The optimal DES, choline chloride–glycerol (ChCl-Gly) with a molar ratio of 1:2 and a 20% water content, was rationally screened using the COSMO-RS model. Subsequent optimization of the extraction parameters was conducted using response surface methodology (RSM). The maximum resveratrol content reached 4.45 ± 0.25 mg/g, which proved similar to that obtained by conventional ethanol reflux extraction (ERE), under the following parameters: 64 °C bath temperature, 200 W ultrasonic power, 20.5 min duration, and 33.3 mL of liquid-to-solid ratio. Notably, the DES-UAE method achieved this efficiency in a significantly shorter time (20.5 min vs. 60 min for ERE). Scanning electron microscopy (SEM) indicated that the combined action of DES and ultrasound caused more severe disruption of plant cell walls than ERE, providing a mechanistic explanation for the enhanced extraction efficiency. This work demonstrates that COSMO-RS-guided screening of DES enables the rapid identification of an optimal solvent system—ChCl-Gly—for the efficient ultrasound-assisted extraction of resveratrol from *P. cuspidatum*. The integration of computational prediction with experimental validation not only accelerated solvent selection but also yielded a fast, effective, and environmentally friendly alternative to conventional extraction methods.

## Highlights

• Rational screening of optimal ChCl-Gly for resveratrol extraction via COSMO-RS predictions.
• Establishment of an efficient UAE-DES process achieving a comparable yield to conventional reflux in one-third of the time.
• SEM visualization reveals synergistic cell wall disruption by DES and ultrasound, explaining the enhanced extraction mechanism.

## 1 Introduction

Resveratrol is a natural polyphenolic compound that was initially extracted from the roots of North American *Veratrum* species, which also gave it its name [1]. As a stress-induced defensive phytochemical, it accumulates in various organs of grape, peanut, berry, *Polygonum cuspidatum*, and mulberry [2]. This compound exhibits significant and broad pharmacological activities, such as antitumor, cardioprotective, antioxidant, antimicrobial, antiviral, hepatoprotective, anti-inflammatory, and immunomodulatory effects [3], [4], [5]. As a dietary supplement, it has drawn interest for its potential to prevent disease and promote wellness. Moreover, resveratrol is recognized for its inhibitory effects on tyrosinase, monophenolase, and diphenolase, contributing to skin-whitening applications, and has been included in China’s *Inventory of Cosmetic Ingredients*. Recent studies have also explored its role in gut microbiota modulation and the management of inflammatory disorders, including colorectal cancer, spurring increased interest in advancing efficient extraction methodologies [6].

In *P. cuspidatum*, resveratrol primarily exists in glycosylated forms, with polydatin being the most abundant. Specifically, polydatin content is 7- to 12-fold higher than that of free resveratrol [7], [8], [9]. Because of this abundance, *P. cuspidatum* is regarded as the preferred raw material for resveratrol production; the central task is therefore to convert polydatin into the bioactive aglycone efficiently. Some extraction techniques—ethanol reflux, microwave-assisted, ultrasound-assisted, and subcritical water extraction—all solubilise the compounds quickly, yet they do little to hydrolyse the glycosidic bond. Consequently, an extra hydrolysis step is required: polydatin must be cleaved to release resveratrol, which is otherwise poorly absorbed in humans [10], [11]. Enzymatic pretreatment with cellulase kills two birds with one stone: it disrupts the cell wall and catalyses the polydatin resveratrol conversion in mild conditions [12], [13]. The drawback, however, is time—enzyme-assisted deep eutectic solvent extraction typically demands several hours [8]. To overcome the rate-limiting kinetics, ultrasound has been coupled with enzymolysis: the acoustic cavitation accelerates mass transfer and shortens reaction time without extra chemicals or heat. This enzyme-plus-ultrasound strategy is now emerging as the most efficient and green route for resveratrol recovery from *P. cuspidatum* [14], [15].

Deep eutectic solvents (DESs), readily prepared by hydrogen-bond pairing of acceptors and donors in defined ratios, are applauded as green, tunable media [16]. When the components are restricted to natural amino acids, polyols, or organic acids, the resulting subclass—termed “natural deep eutectic solvents” (NADES)—combines full biodegradability, recyclability, and water miscibility, offering an ideal platform for the extraction of natural products [17], [18], [19], [20]. Recent studies have shown that acidic DES or the addition of hydrochloric acid can enhance resveratrol extraction from *P. cuspidatum* [21]. However, such acidic systems pose environmental and practical challenges, limiting their green credentials. The rational design of DES is thus crucial for achieving both high extraction efficiency and environmental sustainability. In this context, the Conductor-like Screening Model for Real Solvents (COSMO-RS) has become a powerful computational tool for predicting solute–solvent interactions and guiding the selection of optimal DES compositions before experimental validation [22], [23]. Recent studies have demonstrated that solvent screening via COSMO-RS can markedly shorten development time, reduce solvent consumption, and improve screening efficiency by several-fold [24], [25], [26].

Several DESs were assembled for this study and screened in silico with COSMO-RS model. Choline chloride served as the hydrogen-bond acceptor, while various hydrogen-bond donors were ranked according to their predicted affinity for resveratrol. The top-performing DES were then synthesized and combined with cellulase enzymatic pretreatment under ultrasound assistance to extract resveratrol from *P. cuspidatum*. Following the initial screening of extraction conditions, response surface methodology (RSM) was implemented to fine-tune the process parameters. Moreover, the extraction mechanism was investigated at the molecular level through intermolecular interaction analysis and molecular dynamics simulations, providing theoretical insight into the roles of DES in enhancing the recovery of resveratrol.

## 2 Materials and methods

### 2.1 Materials and instruments

*P. cuspidatum* roots were harvested in Yulin, Shanxi, China (October 2024) and verified by Associate Prof. Xueying Chen (Northeast Agricultural University). After drying, the roots were milled to pass a 40-mesh screen. The powder was suspended in pH 5.0 water (1:20, w/v), and cellulase was added at a concentration of 1.2% (w/w). The slurry was then subjected to ultrasonic treatment (200 W) at 45 °C for 90 min. Following filtration, the residue was dried overnight at 60 °C, finely ground, and stored. Resveratrol standard (> 98%, Lot C16195355) and all analytical-grade DES components (choline chloride, glycerol, propylene glycol, and L-proline) were from Shanghai Maclin Co. HPLC-grade acetonitrile, formic acid, and methanol were supplied by Tianjin Fuyu Fine Chemical Co., and deionized water was prepared with a Milli-Q system (Millipore, Boston, USA).

A KQ-250DB ultrasonic cleaning bath (Kunshan, Jiangsu, China) with a fixed base and annealed transducers operating at 50 kHz was employed. The rectangular bath measured 23.5 cm × 13.3 cm × 10.2 cm internally and delivered up to 250 W. The HPLC system consisted of multiple SHIMADZU manual sample handling units (Shimadzu International Trading Co., Ltd.). Resveratrol was separated on a Diamonsil C18 column (4.6 mm × 150 mm, 5 µm; Dikma Technologies) installed in a Shimadzu HPLC system (LC-20AR pump, SPD-20A UV detector).

### 2.2 Determination of resveratrol content in *P. Cuspidatum*

#### 2.2.1 Preparation of resveratrol standard curve

Resveratrol content and standard curve construction were performed using HPLC according to a previously reported method [27]. The mobile phase was composed of 0.1% formic acid in water (eluent A) and acetonitrile (eluent B). A gradient elution program was executed as follows: isocratic at 30% B from 0 to 10 min; a linear increase to 90% B from 10 to 11 min; isocratic at 90% B from 11 to 15 min; a linear decrease back to 30% B from 15 to 17 min; followed by a 20-minute re-equilibration at 30% B. The analysis was performed with the column temperature maintained at 30 °C, an injection volume of 20 µL, a flow rate of 1.0 mL/min, and detection at 306 nm. Prior to each injection, the column was equilibrated with the initial mobile phase for 20 min.

Resveratrol standard (9 mg) was accurately weighed and transferred to a 5 mL brown volumetric flask. Methanol was added to dissolve the compound and brought to volume, yielding a 1.8 mg/mL stock solution. Subsequent dilutions of this stock were made to prepare standard solutions at 0.03, 0.06, 0.09, 0.12, and 0.15 mg/mL. The standard curve for resveratrol was linear (R^2^ = 0.9978) and expressed by the following equation:

(1) $$Y=2\times 10^{8}X+273740$$

Here, Y and X signify the peak area and the concentration of resveratrol (mg/mL), respectively.

#### 2.2.2 Extraction procedure and content Determination

In this procedure, 0.5 g of enzymatically pre-treated *P. cuspidatum* powder was accurately weighed and mixed with the chosen DES at the predetermined liquid-to-solid ratio. The mixture was subjected to ultrasound-assisted extraction under controlled conditions, with power, temperature, and duration set based on the design of the experiment. Following extraction, the resulting solution was appropriately diluted with methanol. The diluted extract was then centrifuged to separate solid residues, and the supernatant was filtered before HPLC analysis. The standard curve developed in Section 2.2.1 was used to quantify the resveratrol content, which was computed using Eq. (2):

(2) $$Y\text{ie}ld=\frac{c\times v}{M}\left(mg/g\right)$$

where M is the sample's mass (g), v is the solution's volume (mL), and c is the resveratrol concentration (mg/mL).

### 2.3 The screening of *DESs*

#### 2.3.1 Simulation screening using COSMO–RS Modeling

To predict the solubility of resveratrol in diverse deep eutectic solvents (DESs) a priori, this study leveraged the Conductor-like Screening Model for Real Solvents (COSMO-RS) developed by Klamt et al. [28], [29], [30]. As a quantum-chemistry-based thermodynamic methodology, COSMO-RS enables efficient screening of solvent candidates without recourse to extensive experimental data, thereby significantly accelerating the design and selection of optimal solvents for specific applications [31], [32].

Based on a review of published studies on the use of DESs for extracting polyphenolic compounds from botanical matrices, and guided by the principle of natural non-toxicity, a group of DES component systems suitable for resveratrol extraction was selected (Table 1). These systems consist of four hydrogen–bond acceptors (HBAs)—betaine (Bet), choline chloride (ChCl), lactic acid (LA), and L–proline (Pro)—along with eleven hydrogen–bond donors (HBDs): glycerol (Gl), lactic acid (LA), propylene glycol (PG), malic acid (MA), citric acid (CA), fructose (Fru), glucose (Glu), sorbitol (Sor), maltose (Mal), and L–proline (Pro).

**Table 1:** HBA and HBD used in the COSMO-RS screening prediction.

**Number**	**Name**	**CAS**	**Molecular weight (g/mol)**	**σ-surface**
1	Choline chloride	67–48-1	139.62	
2	Glycerol	56–81-5	92.09	
3	Lactic acid	50–21-5	90.08	
4	Propylene glycol	57–55-6	76.09	
5	Citric acid	77–92-9	192.12	
6	Malic acid	6915–15-7	134.09	
7	Glucose	50–99-7	180.16	
8	Fructose	57–48-7	180.16	
9	Sorbitol	50–70-4	182.17	
10	Maltose	69–79-4	342.30	
11	L-Proline	147–85-3	115.13	
12	Betaine	107–43-7	117.15	

All compound structures were retrieved from the PubChem database. Each molecular structure then underwent quantum–chemical calculations via the DMol^3^ package integrated in Materials Studio 2023. Geometries were fully optimized, and single-point energy calculations were subsequently carried out at the GGA/PBE level with a DNP basis set under the COSMO solvation model, which treats the surrounding medium as a perfect conductor [26], [33], [34]. The main output of this step is the σ–profile, denoted as p(σ), depicting the screening charge density (σ) probability distribution on the molecule surface. The σ–profile acts as a unique fingerprint that encodes molecular polarity and hydrogen–bonding capacity. For every DES, the σ-profile was assembled by mole-fraction-weighted averaging of the individual HBA and HBD surface-charge distributions. NADES systems were represented as pseudo-clusters obtained by merging one mole of HBA with one mole of HBD. All subsequent COSMO-RS evaluations were executed in COSMOThermX v18 with the relevant parameter set, yielding the infinite-dilution activity coefficient (γ∞) defined in Eq. (3) as a measure of ideal solute–solvent affinity.

(3) $$\ln\gamma^{\infty }=\frac{\mu_{\text{i}}^{\mathrm{solvent}}-\mu_{\text{i}}^{\mathrm{pure}}}{\mathrm{RT}}$$

γ∞ is the infinite-dilution activity coefficient; μ_i_^solvent^ and μ_i_^pure^ refer to the chemical potentials of resvertrol in the solvent phase and in the pure phase at infinite dilution, respectively. The infinite dilution activity coefficient of resveratrol in each DES system, expressed as its natural logarithm (ln(γ^∞^)), was selected as the key screening metric. According to thermodynamic principles, a lower value of ln(γ^∞^) indicates stronger solute–solvent interactions and thus predicts a greater solubility of the solid solute.

#### 2.3.2 DES preparation and experimental verification

Based on the screening results from the COSMO-RS model, the top three performing deep eutectic solvents (DES) were synthesized. In this study, deep eutectic solvents (DESs) were prepared using choline chloride as the hydrogen bond acceptor (HBA). It was combined with one of three hydrogen bond donors (HBDs)—propylene glycol (PG), glycerol (Gly), or L-proline (Pro)—at a fixed molar ratio of 1:2 (HBA:HBD). The mixtures were heated to 80 °C under continuous magnetic stirring in a round-bottom flask until a clear, homogeneous liquid formed. During stirring, a defined amount of water was introduced to achieve a final water content of 20% (w/w), resulting in a stable, transparent, homogeneous liquid. The COSMO-RS model's theoretical prediction was experimentally validated by determining the resveratrol extraction efficiency from *P. cuspidatum* using the top-performing DES, using the procedure described in Section 2.2.2.

### 2.4 The single-factor experiments

The extraction process was optimized using a ChCl-Gly-based DES (1:2 M ratio, 20% water content) for the recovery of resveratrol using *P. cuspidatum* as the plant source. Four key extraction parameters—time (10–50 min), ultrasonic power (100–250 W), temperature (40–70 °C), and liquid–solid ratio (10–50 mL/g)—were optimized through single-factor experiments. Each factor was systematically varied while keeping others constant to assess its individual effect. The primary objective of this single-factor study was to determine the approximate optimal levels (central points) for the key variables, which then served as the basis for designing a more efficient response surface methodology analysis.

### 2.5 Response surface methodology (RSM) optimization

Guided by the single-factor experimental results, the extraction conditions for resveratrol from *P. cuspidatum* were further optimized using a four-factor, three-level Box-Behnken design (BBD). The independent variables designated for this study were extraction time (X_1_), ultrasonic power (X_2_), extraction temperature (X_3_), and liquid–solid ratio (X_4_). Each factor was assigned three coded levels: −1 (low), 0 (medium), and + 1 (high), with their specific values listed in Table 2. The experimental design comprised a total of 29 runs, including five center points to estimate the pure error. The target variable was the yield of resveratrol. The relationship between the variables and the response was modeled by fitting a second-order polynomial equation to the experimental data:

(4) $$Y=\beta_{0}+\sum_{\text{i}=1}^{4}\beta_{i}X_{i}+\sum_{\text{i}=1}^{4}\beta_{\mathrm{ii}}X_{i}^{2}+\sum_{i<j}^{4}\beta_{\mathrm{ij}}X_{i}X_{j}$$

where Ydenotes the predicted value. The model includes an intercept (β_0_), with β_i_, β_ii_, and β_ij_ representing the coefficients for the linear, quadratic, and interaction terms, respectively.

**Table 2:** Coding of experimental parameters and related levels.

Experimental parameters	Unit	Symbols (X_i_)	Coded values		
			Low (−1)	Medium (0)	High (+1)
Extraction time	min	X_1_	10	20	30
Ultrasonic power	W	X_2_	150	200	250
extraction temperature	°C	X_3_	50	60	70
Liquid-solid ratio	mL/g	X_4_	20	30	40

The significance and adequacy of the developed model were evaluated through ANOVA, the R^2^ value, and a lack-of-fit test. To ensure reliability, each extraction was performed in triplicate. The experimental data were subsequently analyzed using Design-Expert software (Version 13, Stat-Ease Inc., Minneapolis, MN, USA).

### 2.6 Comparison with conventional extraction methods

#### 2.6.1 Comparison of extraction yield with conventional reflux extraction

Extraction efficiency was quantitatively evaluated by comparing the resveratrol yields achieved with the novel method and the conventional ethanol reflux process. In the traditional method, 1 g of *P. cuspidatum* powder was refluxed in 20 mL of an 80% ethanol solution at 80 °C for 1 h. The content of resveratrol in the resultant extracts from both methods was precisely quantified following the process in Section 2.2.2.

#### 2.6.2 Microscopic Comparison of Post-Extraction powders by Scanning electron microscopy (SEM)

The microstructural alterations in the herbal residues induced by two extraction methods were assessed using SEM. Observations were carried out at magnifications ranging from 300 to 1500 times, with the accelerating voltage set at 5.00 kV and the working distance maintained at 13.5 mm. The surface morphology of the powder treated with the conventional ethanol reflux method was compared with that of the powder treated with the UAE of resveratrol from *P. cuspidatum* using DES, as well as with untreated powder. The SEM micrographs provide visual evidence for the disruptive effects on the plant cell walls, thereby elucidating the mechanisms contributing to the differing extraction efficiencies.

### 2.7 Comparison of extraction cycles

Recognizing the need to move beyond single-cycle extraction, this study was designed to quantify the impact of repeated extraction cycles (1–5) on process yield. A comprehensive series of tests was conducted under the optimal and consistent experimental conditions established in our prior work, ensuring a reliable assessment of how cycle number influences extraction efficiency.

### 2.8 Analytical statistics

Statistical significance (*P* < 0.05) was assessed by one-way ANOVA followed by Tukey's test. Meanwhile, the design, optimization, and analysis of the response surface methodology (RSM) were conducted with the aid of Design Expert 13 software; this included evaluating regression models, determining parameter contributions and significance, generating response surface plots, and identifying the optimal extraction conditions.

## 3 Results and discussion

### 3.1 Prediction of resveratrol solubility property by COSMO-RS model

#### 3.1.1 Solubility analysis by COSMO-RS model

**Fig. 1:**
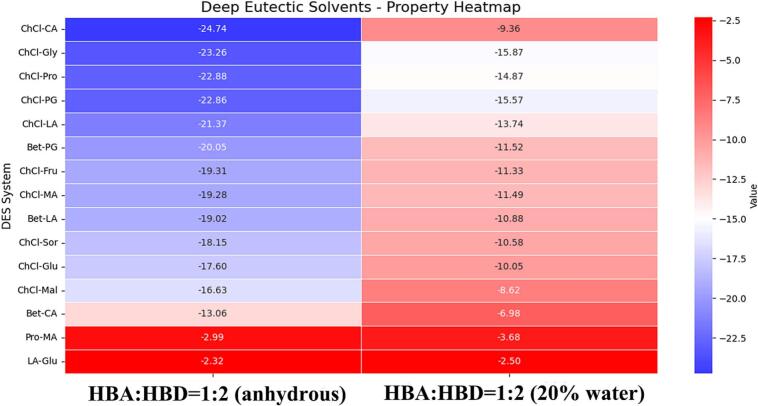
The solubility analysis of resveratrol in DESs. (A) The ln(γ^∞^) selective heat map of resveratrol in DESs (anhydrous). (B) The ln(γ^∞^) selective heat map of resveratrol in DESs (20% water).

The COSMO-RS model was employed to predict the solubility of resveratrol in various deep eutectic solvents (DESs) through calculation of the infinite dilution activity coefficient (ln(γ∞)). The screening results are shown in Fig. 1. A lower ln(γ^∞^) value indicates a stronger affinity between the solvent and resveratrol, and thus a higher theoretical solubility. All DESs in the figure are ranked from low to high ln(γ^∞^), with solvents positioned closer to the top representing better performance.

Fig. 1 showed that DESs based on choline chloride–citric acid (ChCl-CA) generally perform excellently under anhydrous conditions, occupying the top of the ranking (i.e., the optimal region). It is particularly noteworthy that systems such as ChCl-Gly, ChCl-Pro, and ChCl-PG are predicted to have very high solubility for resveratrol. These solvents are typically rich in strong hydrogen bond donors such as hydroxyl and carboxyl groups, which can form an effective hydrogen-bonding network with the phenolic hydroxyl groups of resveratrol. This is likely the main reason for their high dissolution capability. In the presence of 20% water, the solubility of resveratrol in ChCl-CA is predicted to decrease significantly. However, systems such as ChCl-Gly, ChCl-Pro, and ChCl-PG continue to perform excellently, remaining at the top of the ranking. In contrast, DES systems composed of L-proline–malic acid and lactic acid–glucose show relatively high ln(γ^∞^) values (located near the bottom of the figure), indicating limited solubility of resveratrol in these solvents.

Owing to their high viscosity, pure deep eutectic solvents (DESs) are suboptimal for extracting active compounds. To address this, we introduced water as a diluent to lower viscosity and improve extraction performance effectively. Therefore, the ChCl-Gly, ChCl-Pro, and ChCl-PG systems were ultimately chosen as candidate solvents.

#### 3.1.2 σ-surface, σ-profile, and σ-potential analysis

**Fig. 2:**
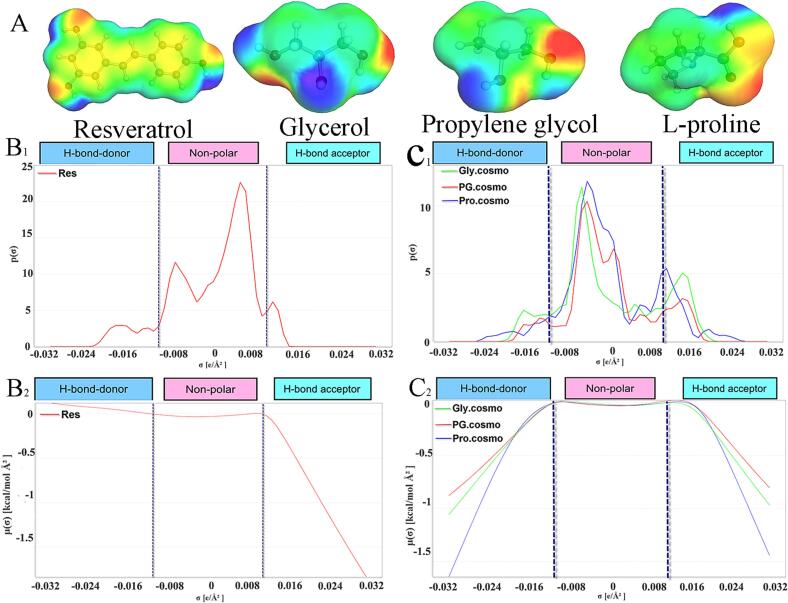
σ-Surfaces (A), σ-profiles (B), and σ-potentials (C) of resveratrol, Gly, PG, and L-Pro.

Fig. 2A presents a COSMO-RS analysis of resveratrol, glycerol, propylene glycol, and L-proline through three complementary descriptors: the σ-potential, σ-profile, and σ-surface. On the σ-surface, the molecular polarity distribution is visualized via a color code: green indicates hydrophobic regions, blue designates hydrogen-bond donor (HBD) sites, and red corresponds to hydrogen-bond acceptor (HBA) sites. The σ-profile represents the probability density distribution of surface segments across different charge densities (σ). Derived from this, the σ-potential indicates a solvent's affinity for a specific σ-interval, where more negative values correspond to stronger attraction. The σ-axis is categorized into three regions: the hydrogen-bond donor (HBD, σ < –0.0082 e Å^−2^), nonpolar (–0.0082 ≤ σ ≤ +0.0082 e Å^−2^), and hydrogen-bond acceptor (HBA, σ > +0.0082 e Å^−2^) regions.

As shown in Fig. 2B1, resveratrol possesses a predominantly hydrophobic surface, as indicated by a distinct peak in the nonpolar region of its σ-profile. In the HBA region, although the probability density is lower, the corresponding σ-potential (Fig. 2B2) decreases sharply, reflecting hydrogen-bonding interactions in the range of 10–40 kJ mol^−1^, which are significantly stronger than van der Waals interactions (1–5 kJ mol^−1^). Thus, despite its large hydrophobic surface, the solubility behavior of resveratrol is primarily governed by hydrogen-bonding interactions in the polar σ-regions.

Analysis of the σ-potential curve (Fig. 2B2) reveals that resveratrol shows a strongly negative potential in the HBA region, while remaining near zero in the HBD region. This suggests that resveratrol primarily serves as a hydrogen-bond acceptor rather than a donor. Therefore, solvents with strong HBD character are expected to form favorable directional hydrogen bonds with resveratrol. According to the σ-complementarity principle, an optimal solvent for resveratrol should provide both adequate nonpolar interaction and strong HBD capability. Comparison of the σ-potentials of the three candidate solvents (Fig. 2C1 and C2) suggests the following solubility enhancement potential: glycerol > propylene glycol > L-proline. This order is consistent with their hydrogen-bond donor strengths and nonpolar matching, as derived from the σ-profiles. It should be emphasized that the present comparison rests on the critical premise that all three DESs share ChCl as the HBA component. Consequently, the σ-profile disparities observed in Fig. 2C—and the inferred solubility-enhancement potential—directly mirror the relative efficiencies with which the different hydrogen-bond donors (Gly, PG, and L-Pro) interact with resveratrol. This confirms that the hydrogen-bond-donating ability of the solvent is critical for dissolving resveratrol.

The COSMO-RS model, as applied in this study, proved to be a robust and efficient tool for the rational preselection of deep eutectic solvents (DES) before experimental validation. Unlike trial-and-error approaches, which are time-consuming and resource-intensive, COSMO-RS enables the rapid evaluation of solute–solvent interactions based solely on molecular structure [35], [36]. The σ-profile and σ-potential analyses provided mechanistic insight into the affinity between resveratrol and various hydrogen bond donors (HBDs). Specifically, the strong negative σ-potential of resveratrol in the hydrogen bond acceptor (HBA) region confirmed its role as a hydrogen bond acceptor, while the positive potential in the nonpolar region indicated hydrophobic compatibility. These findings are consistent with previous studies on polyphenolic compounds such as luteolin and echinacoside, where hydrogen bonding and nonpolar interactions were identified as key drivers of solubility in DES [26], [37].

### 3.2 Experimental validation

**Fig. 3:**
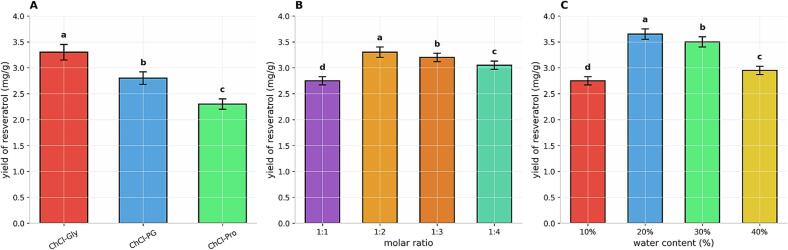
Screening of deep eutectic solvent (DES) systems for ultrasound-assisted extraction (UAE) of resveratrol from *P. cuspidatum*. (A) DES type, (B) molar ratio, and (C) water content. Data are means ± SD, n = 3. Columns bearing different lowercase letters differ significantly (*p* < 0.05); identical letters indicate no significant difference (*p* > 0.05).

Deep eutectic solvents (DES) are composed of varying HBAs and HBDs, which endow them with distinct physicochemical properties. As illustrated in Fig. 3A, these property differences lead to considerable variation in the resveratrol yield among the different DES. Notably, ChCl-Gly demonstrates a significantly higher extraction yield compared to the other solvents. This result aligns well with predictions derived from the COSMO-RS model and σ-potential analysis, thereby validating the reliability of this theoretical approach for rational solvent screening. This result aligns well with predictions derived from the COSMO-RS model and σ-potential analysis, thereby validating the reliability of this theoretical approach for rational solvent screening. DES systems such as ChCl-Gly and ChCl-PG, which ranked highest in the COSMO-RS screening, also exhibited the highest experimental extraction efficiencies. This strong correlation underscores the utility of COSMO-RS in guiding solvent selection for natural product extraction, as similarly reported by Norhidzam et al. [22] and Du et al. [23] for boswellic acid and essential oils, respectively. Importantly, the inclusion of 20% water in the simulation (Fig. 1B) refined the predictions by accounting for viscosity reduction and hydrogen-bond network modulation, which are critical in real extraction systems [38]. This two-step screening strategy—first anhydrous, then hydrated—enhances the practical relevance of COSMO-RS and supports its broader adoption in green solvent design.

Upon identifying ChCl-Gly as the optimal solvent system, the effects of its component molar ratio and water content on the extraction yield were further investigated. Fig. 3B illustrates that the molar ratio of ChCl to Gly significantly influenced the extraction yield under fixed conditions. Within the tested range (HBA: HBD from 1:1 to 1:4), the resveratrol yield initially increased and then decreased with increasing Gly proportion, reaching a maximum at a molar ratio of 1:2. This trend suggests an optimal concentration of hydrogen bond donor (HBD) exists. An appropriate amount of Gly provides sufficient hydrogen-bonding sites for effective resveratrol dissolution, whereas an excessive glycerol ratio may disrupt the stable structure of the DES formed between HBA and HBD or significantly increase viscosity, impairing mass transfer and consequently reducing the extraction efficiency.

Furthermore, water, a common additive in DES, is essential for regulating the physicochemical characteristics of the solvent. As shown in Fig. 3C, the extraction yield exhibited a strong dependence on water content. An initial increase in yield was observed from 10% to 20% water content, which was followed by a gradual decrease beyond this point, with the peak yield occurring at approximately 20%. This enhancement is mainly due to the viscosity reduction of the DES system by moderate water content, which improves fluidity and permeability, hence encouraging the diffusion and disintegration of resveratrol from the plant matrix. Nevertheless, the yield sharply declined as the water content rose above 40%. This decline is likely due to excessive water diluting and disrupting the unique three-dimensional structure of the DES, which is maintained by an intensive hydrogen-bonding network, thereby weakening the crucial interactions with resveratrol molecules and diminishing dissolution capacity.

For the ChCl-Gly system, not only its chemical composition but also the precise molar ratio of its components and the appropriate water content are key parameters determining extraction performance. A molar ratio of 1:2 with 20% water content was identified as optimal, delivering the maximum resveratrol extraction efficiency in this system.

### 3.3 Single-factor experimental results

#### 3.3.1 Effect of extraction time on resveratrol yield

**Fig. 4:**
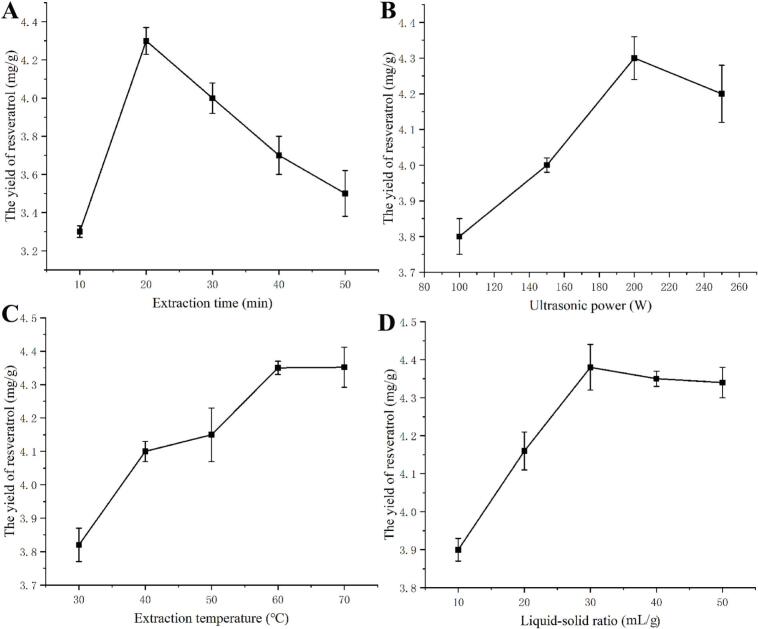
Effects of different parameters on resveratrol yield. (A) ultrasonic time, (B) ultrasonic power, (C) Extraction temperature, (D) Liquid-solid ratio.

Fig. 4A shows the impact of extraction time on resveratrol yield. The yield increases significantly as the extraction duration is extended from 10 min to 20 min. This trend suggests that a longer extraction time allows for more thorough solvent penetration and enhanced mass transfer of the target compound. Nevertheless, when the extraction time is prolonged beyond 20 min to 50 min, the yield begins to decrease gradually. The observed decline may be caused by the prolonged exposure to elevated temperatures, potentially leading to the oxidative degradation of resveratrol. Hence, the optimal extraction time was found to be twenty minutes.

#### 3.3.2 Effect of ultrasonic power on resveratrol yield

The impact of ultrasonic power on resveratrol yield was examined under the optimal conditions described above. Fig. 4B showed that the yield of resveratrol increased and then decreased with increasing ultrasonic power. The highest yield of resveratrol was obtained at 200 W. This is probably because the cavitation effect of ultrasound increases with increasing ultrasonic power, which causes the *P. cuspidatum* cells to be completely broken and facilitates the contact between the solvent and the substrate, thus allowing the resveratrol yield to increase [39]. However, the cavitation effect of ultrasound is too high, causing some molecular structures to be damaged and allowing more impurities to combine with the solvent [40], [41]. Therefore, 200 W was chosen as the focal point for further investigation.

#### 3.3.3 Effect of extraction temperature on resveratrol yield

The extraction temperature exerts a strong influence on the resveratrol yield, as shown in Fig. 4C. The yield increases steadily with increasing temperature (30–60 °C), reaching a maximum value of approximately 4.35 mg/g at 60 °C. This positive correlation can be attributed to enhanced molecular mobility and improved solubility of the target compound at elevated temperatures, which facilitates its diffusion from the plant matrix into the solvent. However, beyond this optimum temperature, the extraction yield remains essentially unchanged. Consequently, 60 °C was chosen as the optimum extraction temperature for subsequent experiments.

#### 3.3.4 Effect of liquid–solid ratio on resveratrol yield

As shown in Fig. 4D, the resveratrol yield increased with the liquid–solid ratio, reaching a maximum at 30 mL/g. However, the resveratrol yield decreased with the increase of the liquid–solid ratio. The reason for this may increases the volume of the extraction solvent, which could enhance mass transfer and facilitate extraction. However, excessive volume could lead to difficulties in subsequent processes, increased energy consumption, and increased loss of resveratrol [42]. Therefore, 30 mL/g was chosen as the focal point for further investigation.

### 3.4 RSM optimization for extracting resveratrol from *P. Cuspidatum*

#### 3.4.1 The model fitting and statistical analysis

A four-factor, three-level design comprising 29 experimental runs was employed to study the effects of extraction time (X_1_), ultrasonic power (X_2_), temperature (X_3_), and liquid-to-solid ratio (X_4_) on resveratrol yield (Y) (Table 3). The experimental data were fitted via multiple regression to a quadratic polynomial model, expressing the response as a function of the independent variables:

**Table 3:** 

Run	Independent variables					The yield of resveratrol (mg/g)	
	X_1_	X_2_	X_3_	X_4_		Actual values	RSM predicted
1	10	150	60	30		2.93	2.88
2	30	150	60	30		2.98	2.89
3	10	250	60	30		2.49	2.57
4	30	250	60	30		2.9	2.95
5	20	200	50	20		2.35	2.33
6	20	200	70	20		3.22	3.11
7	20	200	50	40		2.41	2.4
8	20	200	70	40		3.98	3.89
9	10	200	60	20		2.74	2.8
10	30	200	60	20		2.54	2.62
11	10	200	60	40		2.9	2.83
12	30	200	60	40		3.25	3.32
13	20	150	50	30		2.06	2.26
14	20	250	50	30		2.24	2.16
15	20	150	70	30		3.21	3.42
16	20	250	70	30		3.34	3.27
17	10	200	50	30		2	1.89
18	30	200	50	30		1.93	1.95
19	10	200	70	30		3.03	3.11
20	30	200	70	30		3.02	3.12
21	20	150	60	20		2.98	2.79
22	20	250	60	20		3.03	3
23	20	150	60	40		3.75	3.66
24	20	250	60	40		3.11	3.18
25	20	200	60	30		4.35	4.27
26	20	200	60	30		4.35	4.27
27	20	200	60	30		4.19	4.27
28	20	200	60	30		4.14	4.27
29	20	200	60	30		4.32	4.27

*Note*: X_1_, X_2_, X_3_, and X_4_ denote extraction time (min), ultrasonic power (W), extraction temperature (°C), and liquid-to-solid ratio (mL/g), respectively.

Y = -46.34208 + 0.2765X_1_ + 0.10405X_2_ + 1.06925X_3_ + 0.228917X_4_ + 0.00018X_1_X_2_ + 0.00015X_1_X_3_ + 0.001375X_1_X_4_ − 0.000025X_2_X_3_ − 0.000345X_2_X_4_ + 0.001750X_3_X_4_ − 0.008958X_1_^2^ − 0.000243X_2_^2^ − 0.008858X_3_^2^- 0.004521X_4_^2^.

The established quadratic model was highly significant, as indicated by ANOVA (Table 4) with a p-value < 0.0001 and an F-value of 52.17. This indicates that the model is statistically sound and adept at predicting the resveratrol yield within the experimental domain. The non-significant lack of fit (*p* > 0.05) further validates the model's adequacy in accurately representing the actual factor-response relationship. The linear coefficients in the model for factors X_3_ (temperature) and X_4_ (liquid-to-solid ratio) were both significant factors (*p* < 0.05), underscoring their critical role in the extraction process. In contrast, the linear coefficients for extraction time (X_1_) and ultrasonic power (X_2_) did not show statistical significance at the 5% level. The interaction terms X_2_X_4_ (ultrasonic power and liquid-to-solid ratio) and X_3_X_4_ (extraction temperature and liquid-to-solid ratio), along with all quadratic terms, were extremely significant (*p* < 0.0001). The ANOVA validates the quadratic model as a reliable tool for navigation of the design space. The prominence of interactive and quadratic effects justifies the use of RSM over a simple linear model for identifying optimal extraction conditions for resveratrol from *P. cuspidatum*.

**Table 4:** ANOVA for Quadratic model.

Source	Sum of Squares	df	Mean Square	*F*-value	*P*-value	
**Model**	14.32	14	1.02	52.17	< 0.0001	Significant
X_1_-Extraction time	0.0234	1	0.0234	1.19	0.293	
X_2_-Extraction power	0.0533	1	0.0533	2.72	0.1213	
X_3_-Extraction temperature	3.86	1	3.86	197.14	< 0.0001	
X_4_- liquid–solid ratio	0.5376	1	0.5376	27.42	0.0001	
X_1_X_2_	0.0324	1	0.0324	1.65	0.2194	
X_1_X_3_	0.0009	1	0.0009	0.0459	0.8334	
X_1_X_4_	0.0756	1	0.0756	3.86	0.0697	
X_2_X_3_	0.0006	1	0.0006	0.0319	0.8608	
X_2_X_4_	0.119	1	0.119	6.07	0.0273	
X_3_X_4_	0.1225	1	0.1225	6.25	0.0255	
X_1_2	5.21	1	5.21	265.53	< 0.0001	
X_2_2	2.39	1	2.39	121.94	< 0.0001	
X_3_2	5.09	1	5.09	259.64	< 0.0001	
X_4_2	1.33	1	1.33	67.62	< 0.0001	
**Residual**	0.2745	14	0.0196			
Lack of Fit	0.2359	10	0.0236	2.44	0.2017	Not significant
Pure Error	0.0386	4	0.0096			
**Cor Total**	14.59	28				

#### 3.4.2 Response surface analysis

**Fig. 5:**
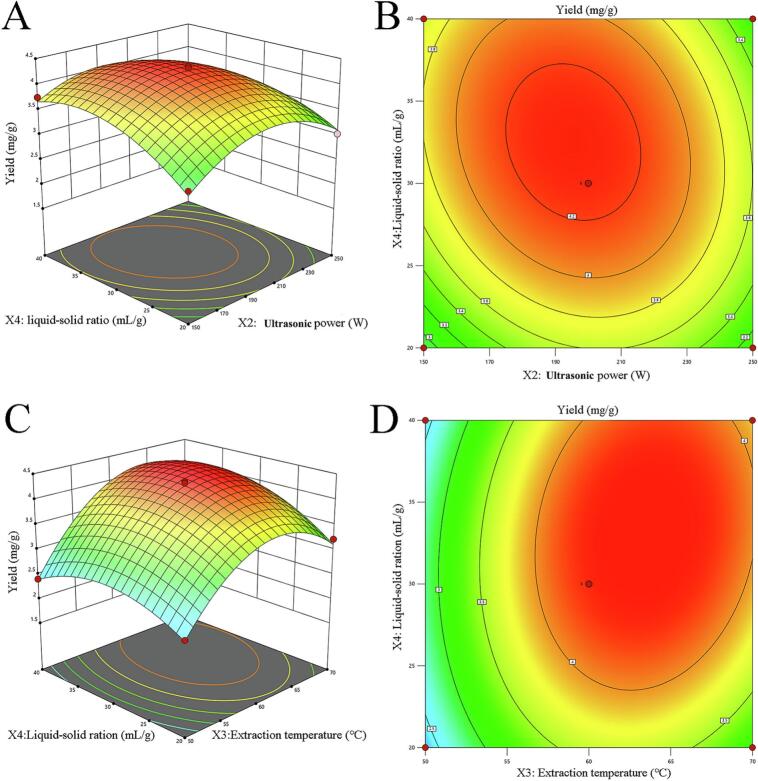
3D response surface graph and contour plot reflect the interactive effects of extraction parameters on the yield of resveratrol from *P. cuspidatum*: Ultrasonic power and Liquid-solid ratio (A, B); extraction temperature and liquid–solid ratio (C, D).

The interactive effects of key process parameters on resveratrol yield are visually interpreted through the generated response surface plots. The three-dimensional surface plot and its corresponding contour plot depicting the interaction between ultrasonic power (X_2_) and liquid-to-solid ratio (X_4_) are shown in Fig. 5A and Fig. 5B. The ANOVA confirmed that this interaction (X_2_X_4_) was highly significant. The pronounced elliptical nature of the contour lines confirms a strong interaction between these two parameters. The response surface exhibits a clear peak, indicating that an optimal combination of liquid-to-solid ratio and ultrasonic power exists for maximizing the yield. The coordinates of this peak correspond well with the predicted optimum values of approximately 195 W and 33.3 mL/g, respectively.

Similarly, the interactive effects of extraction temperature (X_3_) and liquid-to-solid ratio (X_4_) are illustrated in Fig. 5C and Fig. 5D. The significance of the X_3_X_4_ interaction term is reflected in the distinct saddle-shaped or peaked surface of the plot. The yield increases markedly with rising temperature up to an optimum point, beyond which a decline is observed. The contour plot facilitates the identification of a region of maximum yield, centered around 64 °C for temperature and 33.3 mL/g for the liquid-to-solid ratio. The shape of the contours demonstrates that the effect of temperature is more pronounced at certain levels of the liquid-to-solid ratio. Collectively, the analysis of these response surfaces allows for the precise identification of the optimal operational window. The model indicates that maximum resveratrol yield (4.41 mg/g) is obtained with optimal parameters of 20.5 min, 195 W, 64 °C, and a 33.3 mL/g liquid-to-solid ratio.

#### 3.4.3 Validation of the model

The predictive capability of the established model was validated through experiments performed under the identified optimal conditions. The ultrasonic power was adjusted to 200 W to accommodate equipment constraints. The results, summarized in Table 5, show an experimental yield of 4.45 ± 0.24 mg/g, which is in close agreement with the model's prediction of 4.41 mg/g. The strong agreement between the experimental and predicted values validates the robustness of the RSM model and confirms its practical value for optimizing resveratrol extraction from *P. cuspidatum*.

**Table 5:** Predicted and experimental values of the responses at optimum conditions.

Optimum condition					Extraction yield (mg/g)	
Extraction time (min)	Ultrasonic power (W)	Extraction temperature (^◦^C)	Liquid-solid ratio (mL/g)		Predicted value	Experimental value
20.5	200	64	33.3		4.41	4.45 ± 0.24

### 3.5 Compared with traditional methods

#### 3.5.1 Comparison of extraction yield with conventional reflux extraction

**Fig. 6:**
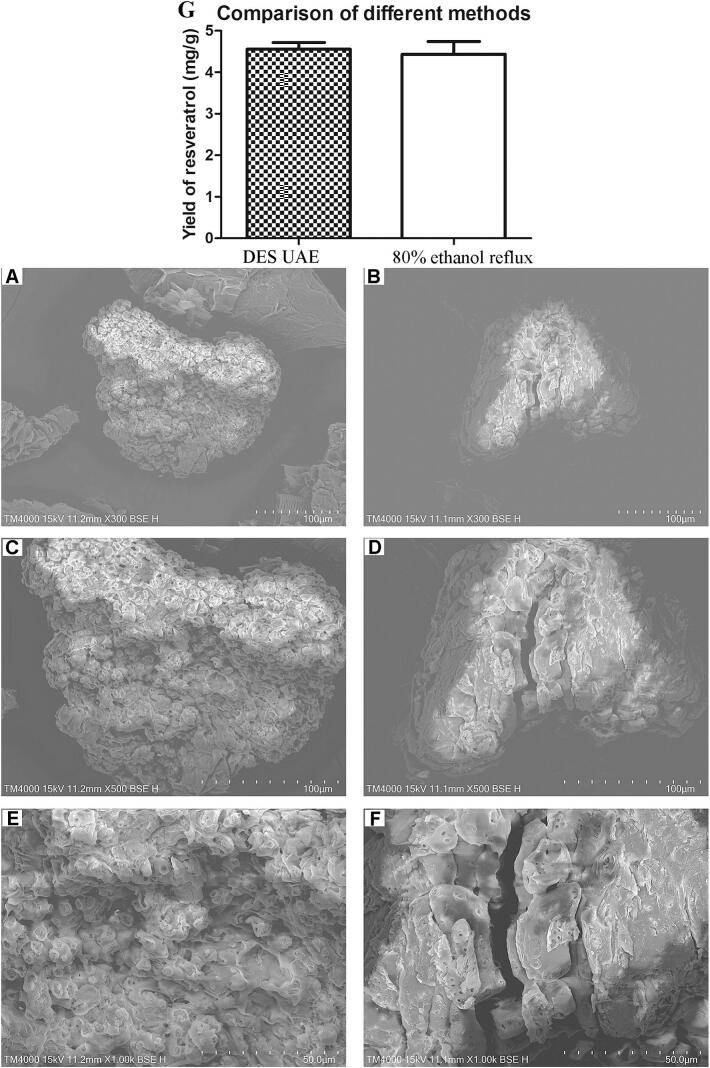
Comparison of effects of UAE and ethanol reflux on resveratrol yield (G) and Scanning Electron Microscopy (SEM) microtopography of *P. cuspidatum* powder. Residue samples derived from DES by UAE (A: × 300; C: × 500; E: × 1500), residue samples derived from 80% ethanol by reflux (B: ×300; D: × 500; F: × 1500).

The extraction efficiency of the optimized UAE process was quantitatively evaluated against the conventional ethanol reflux extraction (ERE) method. As can be seen from Fig. 6G, the resveratrol yield achieved through the optimized UAE process was 4.45 ± 0.24 mg/g, which demonstrated no statistically significant difference from the yield obtained via the conventional ERE method (4.37 ± 0.35 mg/g). Notably, the UAE process achieved this comparable yield within a significantly shorter extraction time (20.5 min) compared to the 60 min required for ERE. This result confirms that the proposed UAE technique can achieve an extraction efficiency comparable to the established benchmark.

The extraction efficiency achieved in this study (4.45 mg/g) using ChCl-Gly-based UAE compares favorably with several recently reported values for resveratrol extraction from *P. cuspidatum*. For instance, a recent study utilizing a betaine-DL-malic acid NADES combined with ultrasound-assisted extraction achieved a resveratrol yield of only 2.95 mg/g (by HPLC analysis), which is substantially lower than the yield obtained in the present work [43]. It is worth noting that some studies have reported higher resveratrol yields (e.g., 12.26 mg/g using acidic DES [21]), but these methods often involve corrosive acids or longer processing times, which may compromise their environmental sustainability. In contrast, the proposed DES-UAE approach achieves a yield comparable to conventional ethanol reflux (4.37 mg/g in this study) while reducing extraction time by two-thirds, lowering energy input, and eliminating volatile organic compounds. This aligns with the growing trend toward green extraction technologies in the pharmaceutical and nutraceutical industries [13], [44].

#### 3.5.2 Microscopic Comparison of Post-Extraction powders by SEM

The morphological characteristics of the residual powders obtained from the deep eutectic solvent-based ultrasonic-assisted extraction (DES-UAE) and the conventional ethanol reflux extraction (ERE) were investigated using scanning electron microscopy (SEM) at sequential magnifications of 300×, 500×, and 1500×, as presented in Fig. 6 (Panels A, C, E for DES-UAE; Panels B, D, F for ERE). This multi-scale analysis provides a comprehensive view of the structural disparities induced by the two distinct extraction mechanisms.

At the macroscopic particle level (300×, Fig. 6A and B), a pronounced difference in particle size and integrity is evident. The DES-UAE powder (Fig. 6A) comprises notably finer and more irregular fragments, forming loose aggregates. This contrasts sharply with the ERE sample (Fig. 6B), where the plant tissue maintains larger, more coherent particle structures. This observation immediately suggests that the combined action of the deep eutectic solvent and ultrasonic cavitation exerts a far greater disruptive force on the plant matrix than the thermal treatment in ethanol.

Progression to higher magnifications (500 × and 1500 × ) elucidates the origin of this macroscopic disparity at the cellular level. The DES-UAE residues (Fig. 6C and E) exhibit a thoroughly disintegrated microstructure. The cell walls are severely ruptured, collapsed, and lack structural continuity, resulting in a landscape of debris with no recognizable cellular organization. This extreme level of destruction can be attributed to a synergistic effect: the deep eutectic solvent likely efficiently disrupts hydrogen bonding and solubilizes structural components, such as lignin and hemicellulose, thereby weakening cell wall integrity. Subsequently, the powerful cavitation forces generated by ultrasound effectively tear apart the pre-weakened matrix, leading to complete mechanical breakdown.

In stark contrast, the ERE residues (Fig. 6D and F) display a well-preserved cellular architecture across the same magnifications. Even at 1500× (Fig. 1F), a continuous, honeycomb-like structure of cell walls is clearly visible. The pores are intact and uniform, indicative of a leaching process in which intracellular contents were dissolved and removed without causing catastrophic physical collapse of the cell wall framework. The damage appears consistent with thermal softening and solvent-mediated extraction, which empties the cells while largely preserving their structural skeletons. The morphological evidence from SEM (Fig. 6) also supports the mechanistic advantage of DES-UAE. The severe disruption of cell walls observed in DES-UAE-treated samples is consistent with findings by Zhao et al. [14] and Qi et al. [19], who attributed enhanced extraction to the synergistic effect of ultrasound cavitation and DES-induced matrix swelling. This physical disruption likely facilitates the release of both free and bound resveratrol, including aglycones derived from polydatin hydrolysis during enzymatic pretreatment.

### 3.6 Comparison of extraction cycles

**Fig. 7:**
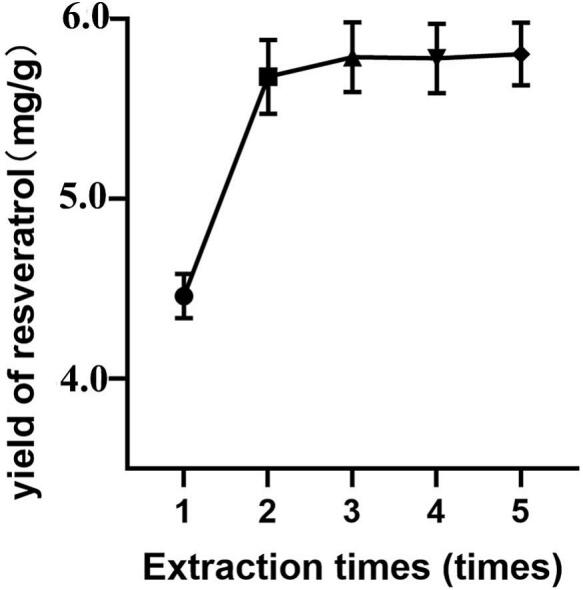
Effects of extraction times on resveratrol yield.

Building on the optimized parameters, this study further examined the influence of extraction cycle number on resveratrol yield. As shown in Fig. 7, the cycle count exerts a pronounced influence. Raising the number of cycles from one to two produced a marked increase in yield (*p* < 0.05), indicating that a single extraction fails to release the target compound completely. Beyond two cycles, however, the yield plateaued, and no statistically significant difference was observed (*p* > 0.05).

This behavior accords with extraction-kinetics principles: the initial concentration gradient is steep, driving strong mass-transfer and a sharp yield rise; as cycles accumulate, the residual solute in the matrix diminishes, the gradient flattens, and subsequent extraction efficiency declines. Moreover, excessive cycles risk solvent waste, higher energy consumption, and potential thermal degradation. Therefore, although marginally higher yields are theoretically attainable with more cycles, overall efficiency, energy use, and economics favor an optimum of two extraction cycles. This finding provides a key benchmark for industrial process optimization.

## 4 Conclusion

This study established a green and highly efficient ultrasound-assisted extraction (UAE) method for resveratrol from *P. cuspidatum* using a deep eutectic solvent (DES) rationally selected via COSMO-RS screening. The optimal solvent system—ChCl-Gly (1:2, 20% water)—was identified based on predicted solute–solvent interactions and experimentally validated. Under conditions optimized by response surface methodology (20.5 min, 200 W, 64 °C, 33.3 mL/g), this approach achieved a resveratrol yield of 4.45 mg/g, which was comparable to conventional ethanol reflux extraction (ERE). Crucially, the developed UAE method reduced the extraction time from 60 min (ERE) to just 20.5 min, representing a three-fold increase in efficiency. SEM analysis further revealed that the synergistic effect of DES and ultrasound caused more severe cell wall disruption than ERE, elucidating the mechanism for the rapid and efficient release of the target compound. The use of COSMO-RS proved instrumental in narrowing down candidate solvents efficiently, avoiding trial-and-error experimentation and highlighting the value of computational tools in green solvent design. Overall, the COSMO-RS-guided DES-UAE strategy offers a rapid, sustainable, and compound-specific alternative for the extraction of resveratrol from plant matrices.

## CRediT authorship contribution statement

**Yitong Lu:** Writing – original draft, Investigation. **Zimeng Song:** Formal analysis. **Pengjun Chen:** Data curation. **Zunlai Sheng:** Writing – review & editing, Funding acquisition, Conceptualization. **Zhiyong Wu:** Conceptualization.

## Declaration of competing interest

The authors declare that they have no known competing financial interests or personal relationships that could have appeared to influence the work reported in this paper.

## References

[1] Vikal A., Maurya R., Bhowmik S., Khare S., Raikwar S., Patel P., Das Kurmi B. (2024). Resveratrol: a comprehensive review of its multifaceted health benefits, mechanisms of action, and potential therapeutic applications in chronic disease. Pharmacol. Res. Nat. Prod..

[2] Ispiryan A., Kraujutiene I., Viskelis J. (2024). Retaining resveratrol content in berries and berry products with agricultural and processing techniques: review. Processes.

[3] Biju B.K., Andavar M. (2025). Resveratrol: potential therapeutic effects on ocular health. Inflammopharmacology.

[4] Islam M.R., Ajaj R., Rauf A., Shanta S.S., Al-Imran M.I.K., Fakir M.N.H., Gianoncelli A., Ribaudo G. (2025). The potential of resveratrol as an anticancer agent: updated overview of mechanisms, applications, and perspectives. Arch. Pharm..

[5] Huang T., Chen X., Chen D., Yu B., Yan H., Zheng P., Luo J., Huang Z. (2026). Comparative study of lipophilicity, cell membrane permeability, and intracellular antioxidant capacity of resveratrol and pterostilbene. J. Nutr. Biochem..

[6] Zheng B., Li R., Chen L. (2024). Control of Starch molecular weight by enzyme treatment facilitates the formation of V-Type starch–resveratrol complexes in a high-pressure homogenization environment and their modulation effects on the gut microbiota. J. Agric. Food Chem..

[7] Sheng H., Zhang B., Shen X., Sun X., Wang J., Yuan Q. (2025). Metabolic engineering of *Escherichia coli* for enhanced biosynthesis of polydatin. Bioresour. Technol..

[8] Li S., Zhang C., Yang R., Zhang Y., Zheng Y., Huang M., Chen D. (2024). Resveratrol production from Polygonum cuspidatum by one-pot green extraction and bioprocessing of polydatin. Ind. Crops Prod..

[9] Chen C.Y., Wang G.H., Kuo J.T., Hsu P.N., Shen Y.C., Chen Y.H., Chung Y.C. (2025). Industrial applications of different parts of flatland polygonum cuspidatum by combining microwave-assisted extraction and fermentation process. Plants.

[10] Renke G., Fuschini A.C., Clivati B., Teixeira L.M., Cuyabano M.L., Erel C.T., Rosado E.L. (2025). New perspectives on the use of resveratrol in the treatment of metabolic and estrogen-dependent conditions through hormonal modulation and anti-inflammatory effects. Curr. Issues Mol. Biol..

[11] Zhao G., Yang L., Zhong W., Hu Y., Tan Y., Ren Z., Ban Q., Yang C.S., Wang Y., Wang Z. (2022). Polydatin, a glycoside of resveratrol, is better than resveratrol in alleviating non-alcoholic fatty liver disease in mice fed a high-fructose diet. Front. Nutr..

[12] He X.-J., Fu J.-X., Jiao J., Gai Q.-Y., Fu Y.-J., Feng X., Wang Y. (2024). Semi-solid-state fermentation of Polygonum cuspidatum roots by a novel endophytic fungus Penicillium rubens with capabilities of cell wall hydrolysis and polydatin deglycosylation to improve the yield of high-value resveratrol. Ind. Crops Prod..

[13] Trombino S., Cassano R., Di Gioia M.L., Aiello F. (2025). Emerging trends in green extraction techniques, chemical modifications, and drug delivery systems for resveratrol. Antioxidants.

[14] H. Zhao, J. Wang, Y. Han, X. Wang, Z. Sheng, 2024. Optimization of process conditions for ionic liquid-based ultrasound-enzyme-assisted extraction of resveratrol from Polygonum Cuspidatum. Ultrason. Sonochem. 108, 106973. doi:10.1016/j.ultsonch.2024.106973. 10.1016/j.ultsonch.2024.106973 PMC11261449 38943848

[15] Xu X., Zhang D., Liu X., Zheng R., Jiang T. (2024). Ultrasonic-assisted extraction and antioxidant evaluation of resveratrol from peanut sprouts. Processes.

[16] Li M., Rao C., Ye X., Wang M., Yang B., Wang C., Guo L., Xiong Y., Cui X. (2022). Applications for natural deep eutectic solvents in chinese herbal medicines. Front. Pharmacol..

[17] Bener M., Şen F.B., Önem A.N., Bekdeşer B., Çelik S.E., Lalikoglu M., Aşçı Y.S., Capanoglu E., Apak R. (2022). Microwave-assisted extraction of antioxidant compounds from by-products of Turkish hazelnut (*Corylus avellana* L.) using natural deep eutectic solvents: modeling, optimization and phenolic characterization. Food Chem..

[18] Wang J.-D., Fu L.-N., Wang L.-T., Cai Z.-H., Wang Y.-Q., Yang Q., Fu Y.-J. (2021). Simultaneous transformation and extraction of resveratrol from Polygonum cuspidatum using acidic natural deep eutectic solvent. Ind. Crops Prod..

[19] Qi H., Fu W., Liu Y., Bai J., Wang R., Zou G., Shen H., Cai Y., Luo A. (2025). Electron beam irradiation coupled ultrasound-assisted natural deep eutectic solvents extraction: a green and efficient extraction strategy for proanthocyanidin from walnut green husk. Food Chem..

[20] Hou Y.-J., Wang P.-W., Zhang H., Fan Y.-Y., Cao X., Luo Y.-Q., Li Q., Njolibimi M., Li W.-J., Hong B., Zhao C.-J. (2024). A high-permeability method for extracting purple yam saponins based on ultrasonic-assisted natural deep eutectic solvent. Food Chem..

[21] Sun B., Zheng Y.L., Yang S.K., Zhang J.R., Cheng X.Y., Ghiladi R., Ma Z., Wang J., Deng W.W. (2021). One-pot method based on deep eutectic solvent for extraction and conversion of polydatin to resveratrol from Polygonum cuspidatum. Food Chem..

[22] Norhidzam M.S., Kai C.W., Hong S.L., Chuah J.H., Yusoff R., Goh B.H., Teoh W.H. (2025). The Design of natural deep eutectic solvent for the extraction of Acetyl-11-Keto-β-Boswellic acid using COSMO-RS. Microchem. J..

[23] Du H., Wang Y., Wu Y., Wu W., Gao B., Yang J., Leng J., Yang M. (2025). Natural deep eutectic solvents combined with COSMO-RS model for efficient extraction of essential oil from Fructus Aurantii: theoretical screening, process optimization, biological activity and mechanism. Microchem. J..

[24] Zhang S., Niu L., Si X., Li L., Sheng Z. (2025). Microwave-assisted extraction of luteolin from peanut shells using natural deep eutectic solvents and its molecular mechanism. Ind. Crops Prod..

[25] Zhuang L., Chen Y., Lei Z., Gui C., Dong Y., Guo Y. (2026). Enhanced solubility of carbamazepine using cholinium-based ionic liquid: from COSMO-RS screening to molecular dynamics simulation. Chem. Eng. Sci..

[26] Zeng Y., Zhong Y., Li S., Deng Y., Ma Y., Huang J., Li H., He Q. (2025). Integration of COSMO-RS and ANN-GA for extraction of echinacoside and acteoside from Cistanche deserticola using natural deep eutectic solvents. Food Chem..

[27] Rabesiaka M., Rakotondramasy-Rabesiaka L., Mabille I., Porte C., Havet J.-L. (2011). Extraction of trans-resveratrol from red wine and optimization by response surface methodology. Sep. Purif. Technol..

[28] Peng W., Wang Y., Wang L., Wang W., Huang J., Zhou R., Bo R., Liu C., Liu M., Li J. (2025). Eco-friendly extraction of polysaccharides from Moringa oleifera Lam. leaves using COSMO-RS screened natural deep eutectic solvents. Food Res. Int..

[29] Peng W., Wang L., Wang X., Wang Y., Wang W., Huang J., Zhou R., Chen C., Bo R., Liu M., Li J. (2025). Efficient polyphenol extraction from Moringa oleifera Lam. leaves using natural deep eutectic solvents: COSMO-RS screening, ANN-GA optimization and antioxidant activity evaluation. LWT.

[30] Aman Z., Khan H.W., Kee L.M., Goto M., Moniruzzaman M. (2025). Solubility Enhancement of valine using deep eutectic Solvents: COSMO-RS and experimental validation. J. Mol. Liq..

[31] Grigorash D., Müller S., Paricaud P., Stenby E.H., Smirnova I., Yan W. (2025). A comprehensive approach to incorporating intermolecular dispersion into the openCOSMO-RS model. Part 1. Halocarbons. Chem. Eng. Sci..

[32] M.A. Escobedo-Monge, S. de-la-Huerta-Sainz, P.A. Marcos, J.A. Esteban-Ollo, L. Montejo-Gil, M. Conde-Rioll, M. Atilhan, A. Bol, S. Aparicio, 2025. Rational Design of Eco-Friendly Deep Eutectic Solvent Systems for the Recovery of High-Value Phytosterol Compounds. Food Bioprocess Technol. 18, 7646 – 7660. doi:10.1007/s11947-025-03896-5.

[33] Klamt A. (2018). The COSMO and COSMO-RS solvation models. WIREs Comput. Mol. Sci..

[34] Andreas K., Frank E., Wolfgang A. (2010). COSMO-RS: an alternative to simulation for calculating thermodynamic properties of liquid mixtures. Annu. Rev. Chem. Biomol. Eng..

[35] Soukup-Carne D., López-Porfiri P., Bragagnolo F.S., Funari C.S., Fan X., González-Miquel M., Esteban J. (2024). Extraction of 5-hydroxymethylfurfural and furfural in aqueous biphasic systems: a COSMO-RS guided approach to greener solvent selection. ACS Sustainable Chem. Eng..

[36] Wang K., Peng D., Alhadid A., Minceva M. (2024). Assessment of COSMO-RS for predicting liquid–liquid equilibrium in systems containing deep eutectic solvents. Ind. Eng. Chem. Res..

[37] Popović B.M., Uka D., Boublia A., Agić D., Kukrić T., Albrahim M., Elboughdiri N., Benguerba Y. (2024). Solubility and extractability enhancement of the main food flavonoids by using choline chloride-based natural deep eutectic solvents. J. Mol. Liq..

[38] Al-Maari M.A., Hizaddin H.F., Hayyan A., Hadj-Kali M.K. (2024). Screening deep eutectic solvents as green extractants for oil from plant seeds based on COSMO-RS model. J. Mol. Liq..

[39] Shen L., Pang S., Zhong M., Sun Y., Qayum A., Liu Y., Rashid A., Xu B., Liang Q., Ma H., Ren X. (2023). A comprehensive review of ultrasonic assisted extraction (UAE) for bioactive components: principles, advantages, equipment, and combined technologies. Ultrason. Sonochem..

[40] Prawira-Atmaja M.I., Puangpraphant S. (2025). Effects of ultrasound-assisted bath, probe, and extraction time on bioactive compounds and antioxidant activity in white tea (*Camellia sinensis*) extracts from purple- and green-leaf cultivars. J. Saudi Soc. Agric. Sci..

[41] Ouyang L., Liang W., Bian C., Shan Y., Wang S. (2025). Ultrasound-assisted green natural deep eutectic solvent extraction of flavonoids from wild blueberry: process optimization, composition identification, and antioxidant activity. Foods.

[42] Xiong X., Yang W., Huang G., Huang H. (2023). Ultrasonic-assisted extraction, characteristics and activity of Ipomoea batatas polysaccharide. Ultrason. Sonochem..

[43] Guo Y., Wan S., Gu Y., He T., Chen Z., Qu X., Quan J., Ma J., Hamid I.A. (2026). Optimization of extraction and antioxidant activities of resveratrol from polygonum cuspidatum by ultrasound-assisted natural deep eutectic solvent method. Molecules.

[44] Bhatia L., Kaladhar D.S.V.G.K., Sarkar T., Jha H., Kumar B. (2024). Food wastes phenolic compounds (PCs): overview of contemporary greener extraction technologies, industrial potential, and its integration into circular bioeconomy. Energy Ecol. Environ..

